# Utilization of tooth filling services by people with disabilities in Taiwan

**DOI:** 10.1186/s12939-016-0347-3

**Published:** 2016-04-05

**Authors:** Ming-Chuan Chen, Pei-Tseng Kung, Hsun-Pi Su, Suh-May Yen, Li-Ting Chiu, Wen-Chen Tsai

**Affiliations:** Department of Public Health, China Medical University, Taichung, Taiwan R.O.C.; Department of Health Services Administration, China Medical University, 91 Hsueh-Shih Road, Taichung, 40402 Taiwan R.O.C.; Department of Healthcare Administration, Central Taiwan University of Science and Technology, Taichung, Taiwan R.O.C.; Department of Healthcare Administration, Asia University, Taichung, Taiwan R.O.C.; Department of Dental Hygiene, China Medical University, Taichung, Taiwan R.O.C.; Department of Chinese Medicine, Nantou Hospital, Nantou, Taiwan R.O.C.

**Keywords:** Tooth filling utilization, Disability, Dental services

## Abstract

**Background:**

The oral condition of people with disabilities has considerable influence on their physical and mental health. However, nationwide surveys regarding this group have not been conducted. For this study, we used the National Health Insurance Research Database to explore the tooth filling utilization among people with disabilities.

**Methods:**

Using the database of the Ministry of the Interior in 2008 which included people with disabilities registered, we merged with the medical claims database in 2008 of the Bureau of National Health Insurance to calculate the tooth filling utilization and to analyze relative factors. We recruited 993,487 people with disabilities as the research sample.

**Results:**

The tooth filling utilization was 17.53 %. The multiple logistic regression result showed that the utilization rate of men was lower than that of women (OR = 0.78, 95 % CI = 0.77–0.79) and older people had lower utilization rates (aged over 75, OR = 0.22, 95 % CI = 0.22–0.23) compared to those under the age of 20. Other factors that significantly influenced the low tooth filling utilization included a low education level, living in less urbanized areas, low economic capacity, dementia, and severe disability.

**Conclusion:**

We identified the factors that influence and decrease the tooth-filling service utilization rate: male sex, old age, low education level, being married, indigenous ethnicity, residing in a low urbanization area, low income, chronic circulatory system diseases, dementia, and severe disabilities. We suggest establishing proper medical care environments for high-risk groups to maintain their quality of life.

## Background

Oral diseases are crucial health concerns for people with disabilities [1]. Because of their physical and mental limitations, the activities of daily living of people with disabilities are restricted. Moreover, people with disabilities have limited self-care abilities, relatively poor health conditions, and a low utilization of medical services [2–4]; hence, they generally have poor oral conditions and several periodontal diseases [5–7]. Oral health conditions generally influence various aspects of life, such as nutritional status, quality of life, and personal perceptions and feelings [8–10]. In addition, oral health is a critical factor influencing overall health. Therefore, the oral health and treatment of people with disabilities require further attention.

Regarding the number of decayed, missing, or filled teeth (i.e., the DMFT index), studies have indicated that the total DMFT score for people with disabilities is higher than that for people without disabilities [6]. Despite the high proportion of decayed or missing teeth, people with disabilities have a low rate of tooth fillings [11]. Tooth decay can induce pain and cause difficulty in food consumption and chewing; in severe cases, infection-caused sepsis can endanger patients’ lives [12, 13]. Missing teeth may alter teeth arrangement, affect chewing ability [14], and trigger periodontal diseases. Moreover, malocclusion caused by missing teeth can induce temporomandibular pain and affect the facial appearance [15].

According to dental early-treatment concepts, tooth fillings can prevent teeth from further decay, preserve the original tooth root, reduce the possibility of severe decay or cavities, and reduce the utilization of additional medical services. Therefore, the utilization of the tooth-filling services is a major indicator of the oral healthcare status for people with disabilities. In addition, the utilization of preventive health services was associated with gender, marital status [16], educational level, age, income, health status, severity of disability, and urbanization level of residence area [17].

This study used a nationwide survey to investigate the tooth-filling service utilization rate among approximately 0.93 million Taiwanese people with disabilities and to explore the factors that influence tooth-filling utilization. Moreover, we present a population-based statistical analysis that could serve as a reference or foundation for future studies.

## Methods

### Data source and processing

We used the database of the Ministry of the Interior (Taiwan) for 2008, which included information of the registered people with disabilities. In 2008, Taiwan had 1,040,585 people with disabilities, accounting for 4.52 % of the population. Additionally, we merged the medical claims database of the Bureau of National Health Insurance of Taiwan for 2008. In 2007, 22.60 million (98.43 %) of Taiwan’s total population (22.96 million) was enrolled in the NHI program. This NHI research database includes the details of all medical services utilized by the enrollees.

We recruited 933,487 participants in 2008. Among the participants, 14,558 (1.56 %) had undergone endodontic therapy, 101,905 (10.92 %) had received amalgam or composite resin tooth fillings, 47,155 (5.05 %) had received both procedures, and 769,869 (82.47 %) did not receive tooth fillings. This study was approved by the institutional review board of China Medical University and Hospital (IRB No. CMUH102-REC3-076).

### Description of variables

The independent variables were (a) personal characteristics, such as sex, age, education level, marital status, and indigenous ethnicity; (b) economic status, such as premium-based monthly salary (seven levels based on income) and low-income households; (c) health condition, such as catastrophic injuries or illnesses, chronic diseases, disability categories, and disability degrees; and (d) environmental factors, such as the degree of urbanization (levels 1–7 for highly urbanized cities and townships, moderately urbanized cities and townships, emerging cities and townships, average cities and townships, aging cities and townships, agricultural cities and townships, and remote cities and townships, respectively).

### Study participants

The study participants encompassed people with disabilities of all ages. Disabilities considered in this study were visual impairment, hearing impairment, speech impediment, limb impairment, mental illness, multiple impairments, major organ malfunction, facial disfigurement, dementia, autism, chromosomal abnormalities, congenital metabolic disorders, congenital defects, mental illness, impaired balance, refractory epilepsy, and disabilities caused by rare diseases. We excluded chronically unconscious patients because they are unsuitable for evaluating the tooth-filling service utilization rate.

### Statistical analyses

We used descriptive statistics to analyze the utilization of tooth-filling services by people with disabilities. By exploring tooth-filling service utilization rates and its correlation with several variables through a bivariate analysis, we summarized all variables as distinguishable-category data. Furthermore, we used SAS software, chi-square test, Fisher exact test, and univariate logistic regression to examine the correlation between variables and tooth-filling service utilization rates. In addition, we used logistic regression to examine the potentially independent effect of demographic and clinical variables on utilization. We also performed the Likelihood Ratio Test to evaluate the validity of the adjusted logistic regression model. The value of -2LL (i.e., -2 times the log likelihood) was used to assess the significance of logistic regression model. The test was significant and implied the validity of the adjusted logistic regression model in our study.

## Results

### Basic characteristics of people with disabilities

Males comprised 58.6 % (*N* = 547,017) of the participants. The average age of the participants was 49.98 years (SD = 18.97). The majority of the participants were either illiterate or possessed an elementary school level education (42.36 %, *N* = *3*95,409). The urbanization degree of nearly 80 % of the participants ranged from 1 to 4. The dependent-population (i.e., children and older adults) were the largest group when stratified by premium-based monthly salary (33.47 %, *N* = *3*12,480).

People with catastrophic injuries or diseases accounted for 31.17 % (*N* = 290,971). People with chronic diseases such as endocrine and congenital metabolic disorders, mental disorders, circulatory system diseases, digestive system diseases, and musculoskeletal system disorders and connective tissue diseases comprised the majority, with each disease accounting for approximately 30 % of the total. Limb impairment was the largest disability category (37.47 %, *N* = 349,790), followed by mental illness, major organ malfunction, and mental illness (approximately 10 % each). Regarding the disability degree, participants with mild and moderate disabilities accounted for 72.04 % (*N* = 672,409) of the study population (Table 1).

**Table 1:** The tooth filling utilization among people with disabilities: basic characteristics and bivariate analysis

					Did not use		Used		
Variables			Total	%	N	%	N	%	*p*-value
Overall rate of use			933,487		769,869	82.47	163,618	17.53	
Gender									<0.001*
	Female		386,470	41.40	312,284	80.80	74,186	19.20	
	Male		547,017	58.60	457,585	83.65	89,432	16.35	
Age									<0.001*
	<20 years		79,290	8.49	58,567	73.86	20,723	26.14	
	20–24 years		30,235	3.24	23,740	78.52	6,495	21.48	
	25–29 years		41,815	4.48	33,296	79.63	8,519	20.37	
	30–34 years		44,714	4.79	36,249	81.07	8,465	18.93	
	35–39 years		53,901	5.77	44,191	81.99	9,710	18.01	
	40–44 years		73,354	7.86	60,246	82.13	13,108	17.87	
	45–49 years		97,836	10.48	80,184	81.96	17,652	18.04	
	50–54 years		98,301	10.53	80,820	82.22	17,481	17.78	
	55–59 years		92,438	9.90	76,513	82.77	15,925	17.23	
	60–64 years		73,382	7.86	61,112	83.28	12,270	16.72	
	65–69 years		81,554	8.74	68,799	84.36	12,755	15.64	
	70–74 years		82,694	8.86	71,648	86.64	11,046	13.36	
	≥75 years		83,973	9.00	74,504	88.72	9,469	11.28	
average age (mean, SD)			49.98	18.97	50.76	18.73	46.27	19.66	
Educational level									<0.001*
	Elementary school and under		395,409	42.36	339,179	85.78	56,230	14.22	
	Junior high school		160,067	17.15	132,981	83.08	27,086	16.92	
	Senior (vocational) high school		170,568	18.27	134,799	79.03	35,769	20.97	
	Junior college and university or above		78,672	8.43	57,953	73.66	20,719	26.34	
	Unclear		128,771	13.79	104,957	81.51	23,814	18.49	
Marital status									<0.001*
	Married		421,340	45.14	348,931	82.81	72,409	17.19	
	Unmarried		235,128	25.19	189,964	80.79	45,164	19.21	
	Divorced or widowed		31,110	3.33	25,891	83.22	5,219	16.78	
	Unclear		245,909	26.34	205,083	83.40	40,826	16.60	
Aboriginal status									<0.001*
	No		915,624	98.09	754,092	82.36	161,532	17.64	
	Yes		17,863	1.91	15,777	88.32	2,086	11.68	
Urbanization of residence area^a^									<0.001*
	Level 1		171,415	18.36	134,078	78.22	37,337	21.78	
	Level 2		239,908	25.70	192,884	80.40	47,024	19.60	
	Level 3		178,416	19.11	147,534	82.69	30,882	17.31	
	Level 4		178,728	19.15	151,507	84.77	27,221	15.23	
	Level 5		36,456	3.91	32,053	87.92	4,403	12.08	
	Level 6		66,598	7.13	58,017	87.12	8,581	12.88	
	Level 7		61,966	6.64	53,796	86.82	8,170	13.18	
Premium-based monthly salary (NT$)									<0.001*
	Dependent population		312,480	33.47	257,249	82.32	55,231	17.68	
	≤17,280		262,965	28.17	220,503	83.85	42,462	16.15	
	17,281–22,800		253,096	27.11	212,029	83.77	41,067	16.23	
	22,801–28,800		35,726	3.83	27,978	78.31	7,748	21.69	
	28,801–36,300		24,772	2.65	19,049	76.90	5,723	23.10	
	36,301–45,800		26,214	2.81	19,857	75.75	6,357	24.25	
	≥45,801		18,234	1.95	13,204	72.41	5,030	27.59	
Low-income household									<0.001*
	No		877,491	94.00	722,409	82.33	155,082	17.67	
	Yes		55,996	6.00	47,460	84.76	8,536	15.24	
Catastrophic injury or disease									<0.001*
	No		642,516	68.83	534,430	83.18	108,086	16.82	
	Yes		290,971	31.17	235,439	80.91	55,532	19.09	
Relevant chronic diseases									
	Cancer								<0.001*
		No	884,359	94.74	730,664	82.62	153,695	17.38	
		Yes	49,128	5.26	39,205	79.80	9,923	20.20	
	Endocrine and metabolic disease								0.001*
		No	664,445	71.18	547,440	82.39	117,005	17.61	
		Yes	269,042	28.82	222,429	82.67	46,613	17.33	
	Mental illness								<0.001*
		No	668,022	71.56	557,214	83.41	110,808	16.59	
		Yes	265,465	28.44	212,655	80.11	52,810	19.89	
	Disease of the nervous system								<0.001*
		No	789,656	84.59	652,148	82.59	137,508	17.41	
		Yes	143,831	15.41	117,721	81.85	26,110	18.15	
	Disease of the circulatory system								<0.001*
		No	589,991	63.20	481,890	81.68	108,101	18.32	
		Yes	343,496	36.80	287,979	83.84	55,517	16.16	
	Disease of the respiratory system								<0.001*
		No	782,423	83.82	649,470	83.01	132,953	16.99	
		Yes	151,064	16.18	120,399	79.70	30,665	20.30	
	Disease of the digestive system								<0.001*
		No	682,929	73.16	568,242	83.21	114,687	16.79	
		Yes	250,558	26.84	201,627	80.47	48,931	19.53	
	Disease of the urinary system								<0.001*
		No	869,390	93.13	716,469	82.41	152,921	17.59	
		Yes	64,097	6.87	53,400	83.31	10,697	16.69	
	Disease of the skeletal and muscular system and connective tissue								<0.001*
		No	692,897	74.23	577,247	83.31	115,650	16.69	
		Yes	240,590	25.77	192,622	80.06	47,968	19.94	
	Disease of the eyes and auxiliary organs								<0.001*
		No	856,755	91.78	709,338	82.79	147,417	17.21	
		Yes	76,732	8.22	60,531	78.89	16,201	21.11	
	Infectious diseases								<0.001*
		No	905,982	97.05	747,844	82.55	158,138	17.45	
		Yes	27,505	2.95	22,025	80.08	5,480	19.92	
	Congenital malformation								<0.001*
		No	912,276	97.73	753,770	82.63	158,506	17.37	
		Yes	21,211	2.27	16,099	75.90	5,112	24.10	
	Skin and subcutaneous tissue disorders								<0.001*
		No	866,628	92.84	716,390	82.66	150,238	17.34	
		Yes	66,859	7.16	53,479	79.99	13,380	20.01	
	Diseases of the blood and blood-forming organs								<0.001*
		No	902,629	96.69	744,849	82.52	157,780	17.48	
		Yes	30,858	3.31	25,020	81.08	5,838	18.92	
	Diseases of the ear and mastoid process								<0.001*
		No	885,954	94.91	732,903	82.72	153,051	17.28	
		Yes	47,533	5.09	36,966	77.77	10,567	22.23	
	Others								<0.001*
		No	821,048	87.95	681,738	83.03	139,310	16.97	
		Yes	112,439	12.05	88,131	78.38	24,308	21.62	
Type of disability									<0.001*
	Visual impairment		46,201	4.95	38,131	82.53	8,070	17.47	
	Hearing impediment		85,252	9.13	67,004	78.60	18,248	21.40	
	Speech impediment		12,539	1.34	10,210	81.43	2,329	18.57	
	Limb impediment		349,790	37.47	292,704	83.68	57,086	16.32	
	Mental retardation		94,627	10.14	78,660	83.13	15,967	16.87	
	Multiple impediments		90,649	9.71	78,549	86.65	12,100	13.35	
	Major organ malfunction		105,927	11.35	85,828	81.03	20,099	18.97	
	Facial disfigurement		4,349	0.47	3,505	80.59	844	19.41	
	Dementia		16,441	1.76	14,766	89.81	1,675	10.19	
	Autism		9,155	0.98	6,484	70.82	2,671	29.18	
	Chromosomal abnormalities		2,102	0.23	1,645	78.26	457	21.74	
	Congenital metabolic disorders		611	0.07	389	63.67	222	36.33	
	Congenital defect		1,106	0.12	836	75.59	270	24.41	
	Mental illness		106,442	11.40	84,672	79.55	21,770	20.45	
	Impaired balance		2,733	0.29	2,284	83.57	449	16.43	
	Refractory epilepsy		4,153	0.44	3,148	75.80	1,005	24.20	
	Rare diseases		1,410	0.15	1,054	74.75	356	25.25	
Severity of disability									<0.001*
	Mild		354,883	38.02	282,016	79.47	72,867	20.53	
	Moderate		317,526	34.02	262,009	82.52	55,517	17.48	
	Severe		158,648	17.00	137,106	86.42	21,542	13.58	
	Very severe		102,430	10.97	88,738	86.63	13,692	13.37	

^a^Level one: the most urbanized areas

*p < 0.05

### Tooth-filling utilization among people with disabilities

In this study, 17.53 % (*N* = 163,618) of the participants used tooth-filling services (Table 1). The tooth-filling service utilization rate for males (16.35 %) was lower than that for females (19.20 %) (*p* <0.001). Younger participants (<20 years) demonstrated a high utilization rate (26.14 %), whereas older participants (>30 years) demonstrated a utilization rate of <20 % (*p* <0.001).

Participants with high education levels had a high tooth-filling service utilization rate, whereas illiterate participants and those with an elementary school level education had the lowest utilization rate of 14.22 % (*p* <0.001). Regarding marital status, unmarried people had a slightly higher utilization rate compared to that of other groups (*p* <0.001). The use of tooth-filling services was higher in highly urbanized areas (21.78 %) compared with that in other areas (<20 %). As the urbanization degree declined, the utilization rates decreased (*p* <0.001). Regarding economic status, participants with a high premium-based monthly salary had a high utilization rate (*p* <0.001), whereas participants from low-income households exhibited a low utilization rate (15.24 %) (*p* <0.001).

Participants with chronic diseases demonstrated a higher tooth-filling service utilization rate compared participants without chronic diseases. Notably, participants with circulatory and urinary system diseases exhibited low utilization rates (*p* <0.001). When stratified by disability categories, participants with congenital metabolic disorders and autism had high utilization rates (36.33 % and 29.18 %, respectively), whereas participants with multiple impairments and dementia exhibited low utilization rates (13.35 % and 10.19 %, respectively) (*p* < 0.001). Regarding disability degree, participants with mild disabilities exhibited higher utilization rate (20.53 %) than did other participants (*p* < 0.001) (Table 1).

### Logistic regression models for tooth-filling service utilization among participants with disabilities

After adjustment for the variables, most correlating factors significant affected the utilization rate, except for (a) chronic diseases of the neural and urinary systems and blood and hematopoiesis diseases, and (b) the disability categories of facial disfigurement, congenital defects, impaired balance, and disabilities caused by rare diseases (Table 2).

**Table 2:** Logistic regression models for tooth filling utilization among people with disabilities

Variables		OR	95 % CI		*p*-value
Gender					
	Female	1	–	–	–
	Male	0.78	0.77	0.79	<0.001*
Age					
	<20 years	1	–	–	–
	20–24 years	0.70	0.67	0.72	<0.001*
	25–29 years	0.59	0.57	0.61	<0.001*
	30–34 years	0.49	0.48	0.51	<0.001*
	35–39 years	0.44	0.42	0.45	<0.001*
	40–44 years	0.42	0.41	0.43	<0.001*
	45–49 years	0.41	0.40	0.43	<0.001*
	50–54 years	0.40	0.38	0.41	<0.001*
	55–59 years	0.38	0.37	0.39	<0.001*
	60–64 years	0.37	0.36	0.39	<0.001*
	65–69 years	0.35	0.34	0.36	<0.001*
	70–74 years	0.29	0.28	0.30	<0.001*
	≥75 years	0.22	0.22	0.23	<0.001*
Educational level					
	Elementary school and under	1	–	–	–
	Junior high school	1.15	1.13	1.17	<0.001*
	Senior (vocational) high school	1.38	1.35	1.40	<0.001*
	Junior college and university or above	1.71	1.67	1.75	<0.001*
	Unclear	1.20	1.18	1.22	<0.001*
Marital status					
	Married	1	–	–	–
	Unmarried	1.16	1.14	1.18	<0.001*
	Divorced or widowed	1.05	1.01	1.08	0.011*
	Unclear	0.95	0.93	0.96	<0.001*
Aboriginal status					
	No	1	–	–	–
	Yes	0.76	0.72	0.80	<0.001*
Urbanization of residence area					
	Level 1	1	–	–	–
	Level 2	0.87	0.86	0.89	<0.001*
	Level 3	0.79	0.77	0.80	<0.001*
	Level 4	0.70	0.69	0.71	<0.001*
	Level 5	0.58	0.56	0.60	<0.001*
	Level 6	0.61	0.59	0.63	<0.001*
	Level 7	0.63	0.61	0.64	<0.001*
Premium-based monthly salary (NT$)					
	≤17,280	1	-	–	–
	Dependent population	1.00	0.98	1.02	0.989
	17,281–22,800	1.10	1.09	1.12	<0.001*
	22,801–28,800	1.28	1.24	1.31	<0.001*
	28,801–36,300	1.34	1.29	1.38	<0.001*
	36,301–45,800	1.42	1.37	1.46	<0.001*
	≥45,801	1.48	1.43	1.54	<0.001*
Low-income household					
	No	1	–	–	–
	Yes	0.97	0.95	0.99	0.038*
Catastrophic injury or disease					
	No	1	–	–	–
	Yes	1.12	1.10	1.13	<0.001*
Relevant chronic diseases					
	Cancer	1.06	1.03	1.09	<0.001*
	Endocrine and metabolic disease	1.02	1.01	1.03	0.024*
	Mental illness	1.12	1.11	1.14	<0.001*
	Disease of the nervous system	1.00	0.98	1.02	0.970
	Disease of the circulatory system	0.95	0.93	0.96	<0.001*
	Disease of the respiratory system	1.14	1.12	1.15	<0.001*
	Disease of the digestive system	1.16	1.14	1.18	<0.001*
	Disease of the urinary system	1.01	0.98	1.04	0.428
	Disease of the skeletal and muscular system and connective tissue	1.29	1.27	1.31	<0.001*
	Disease of the eyes and auxiliary organs	1.23	1.21	1.26	<0.001*
	Infectious diseases	1.09	1.06	1.13	<0.001*
	Congenital malformation	1.05	1.02	1.09	0.003*
	Skin and subcutaneous tissue disorders	1.06	1.04	1.09	<0.001*
	Diseases of the blood and blood-forming organs	1.01	0.98	1.04	0.500
	Diseases of the ear and mastoid process	1.16	1.13	1.19	<0.001*
	Others	1.35	1.32	1.37	<0.001*
Type of disability					
	Visual impairment	1	–	–	–
	Hearing impediment	1.13	1.10	1.16	<0.001*
	Speech impediment	1.43	1.40	1.46	<0.001*
	Limb impediment	1.11	1.06	1.16	<0.001*
	Mental retardation	0.89	0.87	0.91	<0.001*
	Multiple impediments	0.94	0.91	0.96	<0.001*
	Major organ malfunction	1.30	1.27	1.33	<0.001*
	Facial disfigurement	0.97	0.89	1.05	0.391
	Dementia	0.75	0.71	0.79	<0.001*
	Autism	1.05	1.01	1.11	0.045*
	Chromosomal abnormalities	0.81	0.73	0.91	0.001*
	Congenital metabolic disorders	1.64	1.39	1.94	<0.001*
	Congenital defect	1.04	0.90	1.20	0.588
	Mental illness	1.04	1.02	1.07	0.001*
	Impaired balance	0.92	0.83	1.02	0.122
	Refractory epilepsy	1.11	1.03	1.19	0.008*
	Rare diseases	0.96	0.84	1.08	0.470
Severity of disability					
	Mild	1	–	–	–
	Moderate	0.87	0.85	0.88	<0.001*
	Severe	0.66	0.65	0.67	<0.001*
	Very severe	0.57	0.56	0.59	<0.001*

*p < 0.05

After controlling for other variables, the analysis revealed that males used tooth-filling services at 0.78 times the rate that females did (95 % CI [0.77, 0.79], *p* <0.001). Based on the reference group, which comprised participants <20 years, the adjusted odds ratio (OR) revealed a trend of declining tooth-filling service utilization rate with increasing age; participants ≥75 years exhibited a low utilization rate (0.22 times) compared with that of the reference group (95 % CI [0.22, 0.23], *p* <0.001). Utilization rate variation among the considered age groups was approximately 80 %.

The tooth-filling service utilization rate for participants from the least urbanized areas was lower (0.63 times) that that for participants from the most urbanized areas (95 % CI [0.61, 0.64], *p* <0.001). Furthermore, participants with a high premium-based monthly salary exhibited high tooth-filling service utilization rates. The tooth-filling service utilization rate for participants with the highest premium-based salary was higher (1.48 times; 95 % CI [1.43, 1.54], *p* <0.001) than that for the reference group comprised of participants with the lowest premium-based monthly salary (NT$ ≤17,280).

Compared with participants with visual impairment, those with mental retardation, multiple impairments, dementia, and chromosomal abnormalities exhibited significantly lower tooth-filling service utilization rate; service utilization was the lowest among participants with dementia (OR = 0.75, 95 % CI [0.71, 0.79], *p* <0.001). Participants with other disabilities exhibited higher utilization rates than did participants with visual impairment. Participants with congenital metabolic disorders exhibited the highest utilization rates compared with other participants (OR = 1.64, 95 % CI [1.39, 1.94], *p* <0.05). Participants with more severe disability levels exhibited low utilization rates, and the utilization rate for participants with extremely severe disabilities was lower (0.57 times) than that for participants with mild disabilities (95 % CI [0.56, 0.59], *p* <0.001; Table 2).

## Discussion

Chalmers et al. (2011) conducted a survey in 2005 and reported that approximately 60 % of people with intellectual and developmental disabilities visit dentists. In addition, people aged 22–64 years exhibited high tooth-filling service utilization rates (approximately 64 %), and people ≥65 years exhibited the lowest utilization rate (45 %) compared with the other age groups [18]. However, studies have revealed that older people demonstrate a high rate of missing teeth and untreated decay or cavities [19], high total DMFT scores, and low tooth-filling rates [20, 21]. These results are consistent with our finding that older peoples’ tooth-filling service utilization rate is low.

We demonstrated that patients with dementia had the lowest tooth-filling service utilization rate. Dementia progressively increases in severity, gradually impairing cognitive function [22]. People with a low cognitive function score have a four-fold higher tendency of not regularly availing dental services [23].

Because people with extreme disabilities have physical or mental limitations, they face difficulty in availing medical care [24], thus developing poor oral health conditions [4, 25]. Participants with extreme disabilities in this study demonstrated a tooth-filling service utilization rate 43 % lower than that of patients with mild disabilities.

Sex, residential area, and economic status influence the medical services utilization rate. Kung, Tsai, and Li (2012) indicated that females utilize more preventive health services than do males. A study conducted in the United Kingdom [26] reported that females more readily seek consultation for illness. In the present study, we demonstrated that 19.20 % of the female participants used tooth-filling services, which is significantly higher than the utilization by male participants (16.35 %; OR = 0.78, 95 % CI 077, 0.79).

A high urbanization degree is indicative of denser population and higher government expenditures, higher density of hospitals and medical institutes, more media broadcasts of health information, and higher access to disease treatment and prevention resources [27, 28]. We revealed that the tooth-filling service utilization rate significantly increased with the degree of urbanization; by contrast, participants living in the least urbanized areas exhibited 40 % lower utilization rates. Furthermore, economic status influenced people’s medical assistance–seeking attitudes and behavior [29]. Participants with a low premium-based salary and those from low-income households demonstrated low tooth-filling service utilization rates.

The overall tooth-filling service utilization rate for participants in this study was only 17.53 %, whereas that for the general population is 54.7 % [30]. People with disabilities exhibit high tooth damage because of poor oral health conditions and oral hygiene compared with people without disabilities [31, 32]. However, the number of people with disabilities seeking dental services or undergoing early treatments is relatively low [3], worsening the tooth conditions until they visited a dentist. Thus, the probability of tooth extraction was higher than that for tooth filling. Similar results were reported in Rodriguez Vazquez et al. (2002), suggesting that when people with mental illnesses experience tooth decay or cavities, they receive destructive therapies, such as tooth extraction, instead of reforming the tooth appearance and function; this phenomenon causes a high rate of missing teeth among people with disabilities [33].

In this study, we focused only on tooth-filling service utilization in 2008 by people with disabilities. Because doctor visit rates, decay and cavity prevalence, other dental treatment conditions, and personal health behavior were not considered in out analyses, we could not determine the actual proportion of people who required tooth fillings, nor could we compare other risk factors; this is the primary limitation of our study. However, people with disabilities have exhibited a higher incidence rate of untreated tooth decay or cavities compared with those without disabilities [11, 34]. Therefore, our analysis of nationwide data substantially supports future research and additional investigations for identifying the risk factors, thus serving as a foundation for cohort studies. Moreover, this study was a population-based study with a substantial sample size. Because we recruited participants who cannot easily be recruited, such as people with extreme disabilities and communication impediments, our results are not biased by the presence of groups with superior functions.

Since this study had a large population-based sample, it had a high statistical power and might cause some weak-association factors to reach significant level (*p* < 0.05). Thus, we suggest readers to focus on the factors with bigger odds ratios and to interpret the factors with a borderline odds ratio in a more conservative way. We advise to use confidence intervals (CIs) instead of p values in terms of practical importance.

## Conclusion

In this nationwide study, we investigated the tooth-filling service utilization rate among people with disabilities. We identified the factors that influence and decrease the tooth-filling service utilization rate: male sex, old age, low education level, being married, indigenous ethnicity, residing in a low urbanization area, low income, chronic circulatory system diseases, dementia, and severe disabilities.

The association between tooth-filling service utilization and maintenance of oral health among people with disabilities is extremely crucial. We should focus on and provide more assistance to people with disabilities. Moreover, we should establish appropriate welfare policies for protecting the health of people with disabilities, for example, improving accessible environment for dental care, or proving dentists with financial incentives for dental care giving to people with disabilities.

## Abbreviations

CI: confidence interval
DMFT: decayed, missing, or filled teeth
NT$: New Taiwan Dollar
OR: odds ratio
SAS: statistics analysis system
SD: standard deviation
